# The Pathophysiology of Sarcopenic Dysphagia: Consideration of Videofluoroscopic Swallowing Studies

**DOI:** 10.7759/cureus.94231

**Published:** 2025-10-09

**Authors:** Kohei Horikawa, Tomoki Nanto, Yuki Uchiyama, Kazuhisa Domen

**Affiliations:** 1 Department of Rehabilitation Medicine, Hyogo Medical University Hospital, Nishinomiya, JPN; 2 Department of Speech-Language-Hearing Therapy, Faculty of Rehabilitation, Morinomiya University of Medical Sciences, Osaka, JPN; 3 Department of Rehabilitation Medicine, School of Medicine, Hyogo Medical University, Nishinomiya, JPN

**Keywords:** hyoid movement, pathophysiology, pharyngeal constriction ratio, sarcopenic dysphagia, videofluoroscopic swallowing study

## Abstract

Background

The pathophysiology of sarcopenic dysphagia remains insufficiently understood. The aim of this study was to clarify the pathophysiology of sarcopenic dysphagia by analyzing data from videofluoroscopic swallowing studies, focusing on the pharyngeal constriction ratio (PCR) as an objective indicator of pharyngeal contraction strength and on the anterior movement distance of the hyoid (AMDH), which contributes to the opening of the upper esophageal sphincter.

Methods

The study had a cross-sectional design and included 52 patients aged 65 years or older who were deemed to require a videofluoroscopic swallowing study. Data obtained during the swallowing of 5 mL of a moderately thick liquid were used to calculate the pharyngeal constriction ratio and the anterior movement distance of the hyoid. Sarcopenic dysphagia is a swallowing disorder caused by sarcopenia of the whole body, including the swallowing muscles. The patients were classified into a sarcopenic dysphagia group (n = 27) and a control group (no sarcopenia and no dysphagia; n = 25). The pharyngeal constriction ratio and anterior movement distance of the hyoid were compared between the two groups.

Results

The pharyngeal constriction ratio was significantly higher (r = 0.76; p < 0.001), and the anterior movement distance of the hyoid was significantly shorter (r = 0.30; p = 0.031) in the sarcopenic dysphagia group.

Conclusions

The pathophysiology of sarcopenic dysphagia may involve reduced pharyngeal contraction strength and decreased anterior movement distance of the hyoid.

## Introduction

Sarcopenic dysphagia is a swallowing disorder caused by sarcopenia that involves the whole body, including the swallowing muscles, as described in a consensus-based sarcopenia and dysphagia position paper by four professional organizations [1]. However, the pathophysiology of sarcopenic dysphagia is not fully understood. A position paper on sarcopenia and dysphagia hypothesized that the clinical manifestations of dysphagia resulting from the reduced mass and strength of the swallowing muscles may include “pharyngeal residue due to reduced pharyngeal contraction and impaired opening of the upper esophageal sphincter,” suggesting a potential mechanism for sarcopenic dysphagia [1]. To provide effective dysphagia rehabilitation during the limited hospitalization period, it is important to understand the pathophysiology of sarcopenic dysphagia.

Currently, the gold standard for evaluating pharyngeal contraction strength is pharyngeal manometry [2]. However, this test is invasive and thus difficult to perform in the elderly, and cost issues limit the number of facilities that can perform it. To address this problem, Leonard et al. developed the pharyngeal constriction ratio (PCR) as an objective assessment index to replace manometry [3]. PCR is an objective indicator of pharyngeal contraction strength, but its usefulness in patients with sarcopenic dysphagia has not been investigated [3-7].

The anterior movement of the hyoid is known to play a role in the opening of the upper esophageal sphincter, and several studies have analyzed the anterior movement distance of the hyoid (AMDH) using videofluoroscopy (VF) data [8-10]. Nakao reported a significantly shorter anterior movement distance of the laryngeal in a small group of patients with sarcopenic dysphagia [11]. Therefore, it is conceivable that AMDH, which is coordinated with laryngeal motion, may also be reduced in patients with sarcopenic dysphagia. However, this possibility has not been investigated in depth.

We hypothesized that sarcopenic dysphagia is associated with reduced pharyngeal contraction strength and/or decreased AMDH, which may contribute to swallowing dysfunction either independently or in combination. The primary purpose of this study was to clarify the pathophysiology of sarcopenic dysphagia. Specifically, we evaluated PCR and AMDH. Both indices were calculated from VF data and compared between the sarcopenic dysphagia group and the control group (no sarcopenia and no dysphagia).

## Materials and methods

Subjects

This cross-sectional study included patients aged ≥65 years who were hospitalized in or attended a consultation at Hyogo Medical University Hospital between November 22, 2023, and March 19, 2025. The participants were consecutively recruited from patients for whom VF was deemed necessary by their rehabilitation physician. Inclusion criteria were (1) age of ≥65 years and (2) requirement of VF. Exclusion criteria were (1) current or prior history of cerebrovascular disease, neuromuscular disease, head and neck cancer, or recurrent nerve palsy; (2) inability to follow examination instructions due to cognitive decline; and (3) inability to hold the tongue pressure probe because of missing anterior teeth.

Data for the study were collected by four speech-language-hearing therapists. The results of the t-test power analysis showed that a sample size of 52 (26 in each group) met the conditions (effect size of 0.8, power of 0.8, and significance level of 5%). The effect size was estimated from preliminary data collected at our institution (14 patients with sarcopenic dysphagia and eight patients without sarcopenia and dysphagia), in which large between-group differences were observed. The study was performed after obtaining approval from the Ethics Review Committee of Hyogo Medical University (approval number: 4563). The purpose of the study was explained to the subjects verbally and in writing, and their written consent to participate was obtained.

Diagnosis of sarcopenic dysphagia

Sarcopenic dysphagia was diagnosed based on a flowchart developed by a Japanese research group [12]. The latest reference values for grip strength developed by the Asian Working Group for Sarcopenia 2019 were used [13].

Grip strength in the dominant hand was measured twice in the sitting position using a digital grip strength meter (T.K.K.5401, Takei Scientific Instruments, Niigata, Japan), with the higher value recorded [13]. Skeletal muscle mass index (SMI) was measured using a body composition analyzer (InBody S10, InBody Japan, Tokyo, Japan). Maximum tongue pressure (MTP) was measured using a tongue pressure-measuring device (TPM-01, JMS, Hiroshima, Japan). The participants were instructed to lightly hold the hard ring of the tongue pressure probe with the upper and lower incisors and to press the balloon against the palate with the tongue using maximum force. The measurement time was seven seconds, and the average value was taken as the MTP [14]. In this study, maximum jaw-opening force (MJOF) was measured in addition to MTP to assess swallowing-related muscles. MJOF was measured in the seated position using a jaw-opening dynamometer (Jaw-Opening Trainer KT2016, Livito, Tokyo, Japan). The participants were instructed to open their mouths as forcefully as possible. After a practice trial, measurements were taken twice, and the mean value was used for analysis [15]. Swallowing function was assessed using the repetitive saliva swallowing test (normal: >2) [16], the modified water swallow test (normal: >3) [17], the water swallow test (normal: <3), and the functional oral intake scale (normal: >5) [18,19]. In this study, dysphagia was defined as the presence of an abnormal result in any of the tests.

Videofluoroscopic swallowing study

VF was conducted in an upright seated position without postural adjustments such as chin down, head rotation, or reclining position. Patients were instructed to swallow 5 mL of a moderately thick liquid, which was prepared using barium water diluted to 40% and a thickening powder. The viscosity of the thickened liquid was measured using a cone-plate-type simplified viscometer (JOVI, Nutri, Mie, Japan) and confirmed to correspond to the “moderately thick” category as defined in the Japanese Dysphagia Diet 2021 by the Japanese Society of Dysphagia Rehabilitation (JSDR) Dysphagia Diet Committee [20]. The data were recorded from the lateral view at 30 frames per second using a digital video recorder (DIGA DMR-XP200, Panasonic, Osaka, Japan). A steel ball with a diameter of 11 mm was affixed to the inferior margin of the mandible as an analytical marker. The presence or absence of laryngeal penetration and aspiration was assessed during the first swallow.

The control group (no sarcopenia and no dysphagia) underwent VF, which involves radiation exposure, in the following two situations. First, in cases where no abnormalities in swallowing function assessments, such as the repetitive saliva swallowing test, were identified but silent aspiration was suspected or the patient reported symptoms such as pharyngeal discomfort, further investigation was deemed necessary following consultation with the attending physician and rehabilitation physician. Second, the participants who were enrolled in the esophageal cancer study (approval number: 3947) undergo the examination preoperatively as part of the study’s routine evaluation protocol.

Quantitative analysis of VF

Quantitative analysis was performed using the ImageJ software (National Institutes of Health, Bethesda, MD). All VF analyses were conducted by a single experienced speech-language-hearing therapist. Inter-rater reliability and blinding procedures were therefore not applied; this has been explicitly noted in the Limitations section. The PCR, AMDH, and normalized residue ratio scale (NRRS) (valleculae, NRRSv; piriform sinuses, NRRSp) were calculated. When multiple swallows were observed, the first swallow was included in the analysis.

PCR

PCR has been shown to have a significant negative correlation with pharyngeal pressure measured by manometry [3,4] and a significant association with aspiration [5]. Because PCR can be obtained from VF images, it does not require additional invasive testing or costs, making it a clinically useful objective indicator of pharyngeal contraction strength.

**Figure 1:**
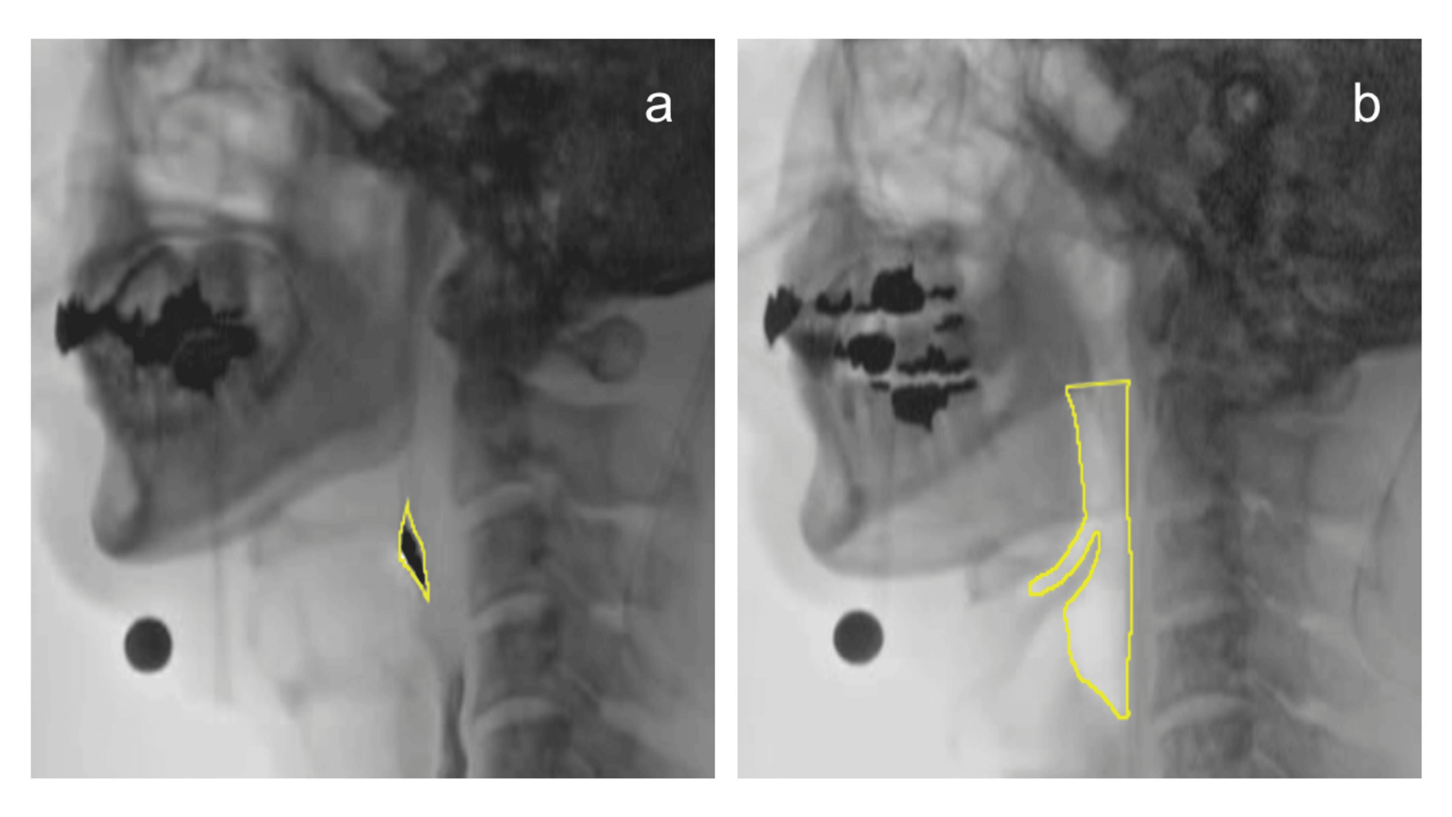
Calculation of the pharyngeal constriction ratio Based on the method developed by Leonard et al., the pharyngeal constriction ratio was calculated by dividing the pharyngeal area at the point of maximum constriction during a swallow (a) by the area with a 1 mL bolus held in the oral cavity (b) [3]

Based on the report by Leonard et al., the pharyngeal area at the point of maximum contraction during swallowing was divided by the pharyngeal area at rest to calculate the PCR (Figure 1) [3]. The pharyngeal area at maximum contraction during swallowing was defined as the frame in which the visible space within the pharynx and the amount of visible bolus were at their minimum. The pharyngeal area at rest was defined as the frame in which 1 mL of moderately thick liquid was being held in the oral cavity. The tracing area for the resting pharyngeal space was defined based on the report by Stokely et al. [2]: superiorly as a line perpendicular to the spine connecting the top of the C2 vertebrae to the tongue base, inferiorly as the base of the pyriform sinuses, posteriorly as the pharyngeal wall, and anteriorly as the wall formed by the base of the tongue and pharyngeal surface of the epiglottis.

AMDH

**Figure 2:**
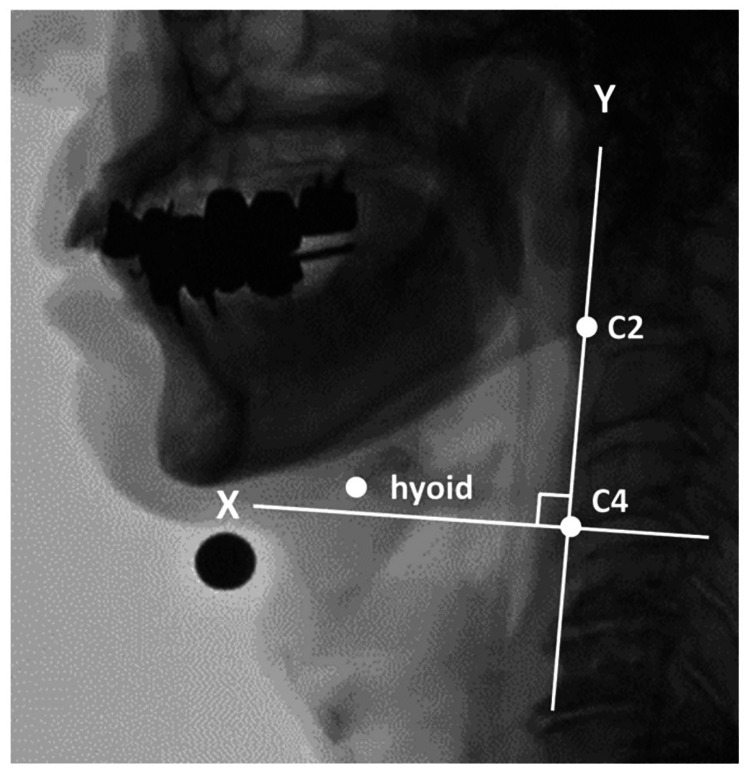
Calculation of the anterior movement distance of the hyoid The resting and maximum anterior positions of the hyoid were plotted to calculate the anterior movement distance of the hyoid. Taking C4 as the origin, the line connecting C2 and C4 is set as the y-axis, and the line passing through C4 and perpendicular to the y-axis is set as the x-axis

The landmarks were defined as the anteroinferior margin of the hyoid, the anterior-inferior corner of the second cervical vertebra (C2), and the anterior-inferior corner of the fourth cervical vertebra (C4) [8]. The resting position of the hyoid and its most anterior position were plotted. The reference axes were defined with C4 as the origin: the line connecting C2 and C4 was designated as the y-axis, and the x-axis was defined as a line perpendicular to the y-axis passing through C4 (Figure 2). A steel ball with a diameter of 11 mm was affixed to the lower border of the mandible and used as a reference for correcting actual distance measurements. In this study, the maximum displacement of the hyoid in the x-axis direction from its resting position was defined as the AMDH.

NRRS

NRRSv and NRRSp were calculated in accordance with the method described by Pearson et al. [21]. Pharyngeal residue was evaluated using the frame in which the hyoid was at its lowest position after swallowing, the glottis had returned to its vertical position, and the pharynx was in a relaxed state. In cases where multiple swallows were observed, the frame in which the hyoid reached its lowest position following the first swallow was used for evaluation. Individual differences were corrected by the square of the distance from C2 to C4.

Statistical analysis

Basic characteristics and each evaluation item were compared between the sarcopenic dysphagia group and the control group. Sex and the presence or absence of laryngeal penetration were analyzed using Fisher’s exact test. Other parameters were analyzed using an independent t-test when normality was confirmed and the Wilcoxon rank sum test when normality was not confirmed. The correlations of PCR and AMDH with NRRSv, NRRSp, MTP, and MJOF were examined in the sarcopenic dysphagia group. Pearson’s correlation coefficient was used for the analysis when normality was confirmed and Spearman’s rank correlation coefficient when it was not confirmed. The statistical analysis was performed using R software (version 3.5.1) (R Foundation for Statistical Computing, Vienna, Austria). A significance level of 5% was adopted.

## Results

Patient characteristics

The patient characteristics are shown for both groups in Table 1. The sarcopenic dysphagia group included 27 patients (mean age: 81.3 ± 6.3 years), in whom the most common major diseases were respiratory diseases (n = 14), cardiac diseases (n = 5), cancer (n = 5), and others (n = 3). The control group included 25 patients (mean age: 75.7 ± 5.4 years), in whom the main diseases were cancer (n = 11), respiratory diseases (n = 10), cardiac diseases (n = 3), and others (n = 1). The mean age was significantly higher in the sarcopenic dysphagia group (p < 0.001), indicating that age may act as a potential confounder in the interpretation of group differences. Although there was no significant between-group difference in sex distribution, the proportion of male patients was higher in both groups. All sarcopenia-related assessment measures, namely, SMI, handgrip strength, MTP, and MJOF, were significantly higher in the control group (all p < 0.001). Significant differences were observed between the two groups in the three swallowing function assessment measures, namely, the functional oral intake scale (p < 0.001), repetitive saliva swallowing test (p < 0.01), and modified water swallow test (p < 0.001). In the sarcopenic dysphagia group, the water swallow test was performed only in patients who showed no abnormalities on the modified water swallow test, in consideration of the risk. As a result, 15 of 27 patients in the sarcopenic dysphagia group underwent the water swallow test, and a significant difference was found between the control group (p < 0.001).

**Table 1:** Patient characteristics according to study group Data are presented as the mean ± standard deviation or median (interquartile range) and as N (%) for categorical variables ^a^Independent t-test (all independent t-tests share the same degrees of freedom {df = 50}; test statistic shown as t) ^b^Fisher’s exact test (no test statistic; indicated as “-”) ^c^Wilcoxon rank sum test (test statistic shown as W) FOIS, functional oral intake scale; MJOF, maximum jaw-opening force; MTP, maximum tongue pressure; SMI, skeletal muscle mass index; MWST, modified water swallow test; RSST, repetitive saliva swallowing test; WST, water swallow test

	Sarcopenic dysphagia (n = 27)	Control (n = 25)	Test statistic	P-value
Age (years)	81.3 ± 6.3	75.7 ± 5.4	t = 3.38	<0.001^a^
Male, n (%)	26 (96.3)	23 (92.0)	-	0.623^b^
SMI (kg/m^2^)	5.50 ± 0.90	6.71 ± 0.87	t = -4.85	<0.001^a^
Handgrip strength (kg)	15.3 ± 5.8	28.6 ± 6.7	t = -7.72	<0.001^a^
MTP (kPa)	19.6 ± 6.1	31.3 ± 6.9	t = -6.46	<0.001^a^
MJOF (kg)	4.4 (3.4, 5.5)	7.7 (5.1, 9.8)	W = 158	<0.001^c^
FOIS	5 (4, 5)	7 (6, 7)	W = 16	<0.001^c^
RSST	2 (2, 4)	4 (3, 6)	W = 178	<0.01^c^
MWST	4 (3, 4)	5 (4, 5)	W = 120	<0.001^c^
WST	3 (3, 4)	2 (1, 2)	W = 367	<0.001^c^

Group comparison of VF parameters

Table 2 compares the VF parameters between the two groups. PCR was significantly higher in the sarcopenic dysphagia group (r = 0.76; p < 0.001). AMDH was significantly shorter in the sarcopenic dysphagia group (r = 0.30; p = 0.031). NRRSv and NRRSp values were significantly higher in the sarcopenic dysphagia group (NRRSv: r = 0.64 and p < 0.001; NRRSp: r = 0.51 and p < 0.001). Aspiration was not observed in any participant in either group. The incidence of laryngeal penetration was significantly higher in the sarcopenic dysphagia group (p = 0.023).

**Table 2:** Comparison of VF parameters between the two groups Data are shown as the median (interquartile range) and as N (%) for categorical variables. Effect sizes are denoted by r for the Wilcoxon rank sum test and by φ for 2 × 2 categorical variables (Fisher’s exact test) ^a^Wilcoxon rank sum test (test statistic shown as W) ^b^Fisher’s exact test (no test statistic; indicated as “-”) AMDH, anterior movement distance of the hyoid; NRRSp, normalized residue ratio scale for the piriform sinuses; NRRSv, normalized residue ratio scale for the valleculae; PCR, pharyngeal constriction ratio; VF, videofluoroscopy

	Sarcopenic dysphagia (n = 27)	Control (n = 25)	Test statistic	Effect size	P-value
PCR	0.26 (0.17, 0.36)	0.03 (0, 0.05)	W = 636	r = 0.76	<0.001^a^
AMDH (mm)	7.14 (4.60, 8.30)	8.83 (7.22, 12.31)	W = 221	r = 0.30	0.031^a^
NRRSv	0.3 (0, 0.47)	0	W = 563	r = 0.64	<0.001^a^
NRRSp	0.13 (0, 0.42)	0	W = 507	r = 0.51	<0.001^a^
Penetration, n (%)	22 (81)	0 (0)	-	φ = 0.82	0.023^b^

Association between PCR, AMDH, and each parameter in the sarcopenic dysphagia group

Table 3 presents the results of the correlation analysis for PCR, AMDH, and each evaluation parameter. PCR showed significant positive correlations with NRRSv (r = 0.71; p < 0.001) and NRRSp (r = 0.39; p = 0.047). In contrast, there was no significant correlation between PCR and MTP (r = -0.28; p = 0.15) or MJOF (r = 0.03; p = 0.89). AMDH did not show a significant correlation with NRRSv (r = 0.01; p = 0.97), NRRSp (r = -0.24; p = 0.22), MTP (r = 0.06; p = 0.78), or MJOF (r = -0.04; p = 0.84).

**Table 3:** Association between PCR and other evaluation parameters in the sarcopenic dysphagia group ^a^Spearman’s rank correlation rho ^b^Pearson’s product-moment correlation MJOF, maximum jaw-opening force; MTP, maximum tongue pressure; NRRSp, normalized residue ratio scale for the piriform sinuses; NRRSv, normalized residue ratio scale for the valleculae; PCR, pharyngeal constriction ratio

	Correlation coefficient (r)	P-value
NRRSv	0.71	<0.001^a^
NRRSp	0.39	0.047^a^
MTP	-0.28	0.15^b^
MJOF	0.03	0.89^a^

## Discussion

In this study, VF data were used to analyze the PCR, an objective indicator of pharyngeal contraction strength, and the AMDH, which is involved in the opening of the esophageal inlet, in order to investigate the pathophysiology of sarcopenic dysphagia.

PCR was significantly higher in the sarcopenic dysphagia group and showed a significant positive correlation with both NRRSv and NRRSp. This finding supports the hypothesis of this study and aligns with a report by Kunieda et al., suggesting that pharyngeal constriction strength is reduced in elderly patients with dysphagia accompanied by sarcopenia [22]. Therefore, the pathophysiology of sarcopenic dysphagia may involve a decrease in pharyngeal constriction strength. However, although the incidence of laryngeal penetration was significantly higher in our sarcopenic dysphagia group, no aspiration was observed. A previous study of the relationship between PCR and aspiration found a significant association between PCR and the occurrence of aspiration in VF [5]. While that study did not specify the bolus type or volume analyzed, it used the largest bolus as the measurement target. In the present study, analysis was limited to a 5 mL bolus to minimize aspiration risk, which may partly explain why no aspiration was observed. Currently, MTP is the most commonly used parameter to assess the strength of the swallowing muscles in patients with sarcopenic dysphagia.

However, this study found no significant correlation between MTP and PCR. Considering that the pharyngeal muscles involved in pharyngeal constriction are also controlled by the respiratory centers and are active in coordination with breathing even outside of swallowing activity [23], it has been suggested that they are less susceptible to disuse atrophy than are the somatic or tongue muscles [22]. Given that our sarcopenic dysphagia group included a substantial number of patients with sarcopenia as a result of disuse, differences in embryological characteristics between the pharyngeal muscles and tongue muscles may have contributed to the lack of correlation between PCR and MTP. Several previous studies have reported a significant association between decreased MTP and sarcopenia [24-28]. However, a study that investigated the relationship between MTP and pharyngeal swallowing pressure using high-resolution manometry found no significant correlation, suggesting that MTP should be considered independently of the severity of sarcopenic dysphagia [22]. Therefore, while MTP is currently used as a representative indicator of the strength of the swallowing muscles when evaluating patients with sarcopenic dysphagia, it is important to recognize that its influence on the pharyngeal stage of swallowing may not be consistent [12]. Looking ahead, reports on the prevention of sarcopenic dysphagia and therapeutic effects are anticipated. PCR may be particularly useful for the investigation of the effectiveness of training methods aimed at enhancing pharyngeal constriction, such as the tongue-hold swallow maneuver.

Hyoid movement during swallowing contributes to the protection of the airway and the transfer of the bolus into the esophagus [9]. Specifically, the superior movement of the hyoid is associated with the closure of the laryngeal vestibule, while anterior movement is more closely related to the opening of the esophageal entrance [8]. In the present study, AMDH was significantly shorter in the sarcopenic dysphagia group than in the control group. This result suggests that the anterior movement distance of the hyoid, which facilitates the opening of the esophageal entrance, may be reduced in association with sarcopenia, supporting the hypothesis mentioned in the position paper on sarcopenia and dysphagia [1]. However, we found no significant correlations between AMDH and NRRS, MTP, or MJOF in our sarcopenic dysphagia group. According to the mechanism of the onset of sarcopenic dysphagia, a decline in hyoid movement may occur even before the clinical manifestation of dysphagia [29]. Considering this mechanism, AMDH may need to be considered independently from the severity of sarcopenic dysphagia.

This study has several limitations. First, the participants did not strictly constitute a consecutive sample, which may have introduced selection bias and requires caution in generalizing the results. This was partly due to staffing circumstances at our institution, as data collection was conducted by four of six speech-language-hearing therapists, without the involvement of two newly employed staff members. Second, the mean age of the sarcopenic dysphagia group was significantly higher than that of the control group, and we cannot rule out the possibility that this age difference influenced the results. Wakabayashi et al. reported that sarcopenic dysphagia was most frequently observed in individuals aged ≥80 years, which is consistent with our findings [30]. Third, although the required sample size was determined by a priori power analysis and met statistical conditions, the overall number of participants was relatively small for a clinical study. This may restrict the external validity and generalizability of the findings. Fourth, all VF analyses were conducted by a single experienced speech-language-hearing therapist. Inter-rater reliability and blinding procedures were not applied, which may introduce assessment bias. Fifth, the analysis of VF data was based on a single swallowing movement with a fixed bolus volume of 5 mL. Data are usually collected multiple times and averaged, but because this was a clinical study, we minimized the risks to the participants by limiting radiation exposure and the potential for aspiration during VF. Nevertheless, the restriction to a single swallow and bolus volume may limit generalizability to broader swallowing conditions. Finally, the PCR and NRRS calculations were based on lateral views, meaning that three-dimensional phenomena were represented in two dimensions. In particular, NRRS cannot capture laterality in residue, which must be taken into consideration. To strengthen the validity and generalizability of these findings, future studies with larger sample sizes and longitudinal designs will be particularly important.

## Conclusions

In this study, we analyzed PCR, an objective indicator of pharyngeal constriction strength, and AMDH, which is involved in the opening of the esophageal entrance, using VF data widely available in clinical practice. Significant differences in PCR and AMDH between the sarcopenic dysphagia and control groups suggest that reduced pharyngeal contraction strength and decreased anterior hyoid movement may reflect the pathophysiology of sarcopenic dysphagia.

It is expected that by comparing dysphagia caused by diseases other than sarcopenia, it will be possible in the future to confirm whether reduced pharyngeal constriction and reduced anterior movement distance of the hyoid are characteristic pathological features of sarcopenic dysphagia. Furthermore, PCR and AMDH, which can be calculated from routinely obtained VF data, may potentially contribute to future assessments and rehabilitation in patients with sarcopenic dysphagia.
